# Percutaneous Temporary Mechanical Circulatory Support as a Bridge to Heart Transplantation in the Current UNOS Allocation System

**DOI:** 10.3390/biomedicines13112637

**Published:** 2025-10-28

**Authors:** Rohan Goswami, Jose Ruiz, Aarti Desai, Peter Wlodkowski, Basar Sareyyupoglu, Sean Kiley, Anirban Bhattacharyya, Daniel Yip, Melissa Lyle, Jose Nativi-Nicolau, Juan Leoni, Devang Sanghavi, Alfredo Quiñones-Hinojosa, Sanjay Chaudhary, Kevin Landolfo, Si Pham, Parag Patel

**Affiliations:** 1 Division of Heart Failure and Transplant, Mayo Clinic, Jacksonville, FL 32224, USA; ruizmorales.jose@mayo.edu (J.R.); desai.aarti@mayo.edu (A.D.);; 2 Department of Cardiothoracic Surgery, Mayo Clinic, Jacksonville, FL 32224, USA; 3 Department of Critical Care Medicine, Mayo Clinic, Jacksonville, FL 32224, USA

**Keywords:** heart transplant, bridge to transplant, mechanical support, heart failure, recovery, cardiomyopathy

## Abstract

**Background**: Progressive heart failure cardiogenic shock (HFCS) often requires escalation to temporary or durable mechanical circulatory support (MCS) as a bridge to transplant (BTT). Following the 2018 UNOS allocation changes, our center revised its BTT strategy to optimize support and shorten wait times. At our institution, the Impella 5.5 with SmartAssist via the axillary approach was selectively used for patients who remained refractory to guideline-directed medical therapy, failed single-inotrope therapy, and were not considered suitable durable LVAD candidates by our multidisciplinary heart team. We compared transplant-related outcomes of BTT patients supported with Impella 5.5 versus durable LVAD. **Methods**: We performed a single-center retrospective review of all heart and heart/kidney transplant candidates at Mayo Clinic Florida from October 2018 to February 2021. INTERMACS profile, baseline characteristics, and perioperative data were collected at the time of device implantation and throughout the transplant hospitalization. **Results**: A total of 87 heart and 4 heart–kidney transplants were completed. Forty-five patients (49%) required MCS as BTT: 27 (60%) with a durable LVAD and 18 (40%) with an Impella 5.5. All eighteen patients with Impella 5.5 as BTT (100%) were transplanted compared to nineteen patients with durable LVAD (70%), *p* = 0.001. The median time from listing to transplant was substantially shorter with Impella (32 vs. 696 days, *p* < 0.001), and this difference persisted across INTERMACS profiles. UNOS status at transplant was more urgent for Impella than LVAD (*p* < 0.001). Transplant surgery following Impella support required shorter cardiopulmonary bypass time (181 vs. 219 min, *p* < 0.001) and resulted in lower postoperative vasoactive-inotropic requirements (7.9 vs. 13, *p* = 0.003). No patients in the Impella group died or were delisted while awaiting transplant, whereas 5 LVAD patients (26%) died or were removed due to LVAD complications (*p* < 0.001). **Conclusions**: Our data demonstrates that the use of the Impella 5.5 as BTT was associated with significantly shorter waitlist time, higher transplantation rates, reduced perioperative morbidity, and lower postoperative vasoactive support compared with durable LVAD as BTT. These benefits were achieved despite a higher severity of illness at transplantation in the Impella cohort.

## 1. Introduction

Over the past decade, the cases of cardiogenic shock (CS) unrelated to myocardial infarction has tripled [1]. With the increase in heart failure cardiogenic shock (HFCS) patients, evaluating the options for bridge therapy is imperative. Given the shortage of donor organs for heart transplantation, left ventricular assist devices (LVAD) have traditionally been considered viable for supporting HFCS patients. Before the changes in the United Network for Organ Sharing (UNOS) heart allocation system in October 2018, LVAD as a bridge to transplant (BTT) was found to have increased rates of transplantation due to UNOS status upgrade-related complications such as stroke, Gastrointestinal (GI) bleeding, allosensitization, and progressive right ventricular (RV) dysfunction [2,3,4].

We found an increased waitlist time within our institution when comparing those patients utilizing LVAD as BTT before October 2018 (497 days) versus after the UNOS change (688 days). Limited data in small case series have shown positive trends in heart transplant recipients bridged to transplantation with the Impella 5.5. Furthermore, recent database reviews demonstrate increased Impella utilization after the 2018 UNOS allocation change without significantly impacting post-transplant survival [5].

## 2. Materials and Methods

A single-center retrospective review was completed of all patients listed for heart or dual organ (heart and kidney) transplantation between 18 October 2018, and 28 February 2021, at Mayo Clinic in Florida after IRB approval. INTERMACS score and baseline patient characteristics were extracted from the medical record based on the LVAD or Impella 5.5 implantation date. Perioperative data for all patients were extracted from electronic medical records during their transplant episode of care. In terms of outcomes, perioperative complications are defined as the pre-transplant occurrence of stroke, device-related infection, allosensitization, and pump thrombosis, GI bleeding that requires more than four units of pRBC transfusions, and progressive RV dysfunction despite inotrope support as measured by right heart catheterization or need for additional temporary mechanical circulatory support (tMCS) devices.

Statistical analysis was performed using SPSS v27 utilizing the Wilcoxon–Mann–Whitney test to determine the significance between the two groups with a *p*-value of <0.05. Multivariate linear regression was performed to model factors affecting survival to transplantation.

### 2.1. Patient Assessment

**Figure 1:**
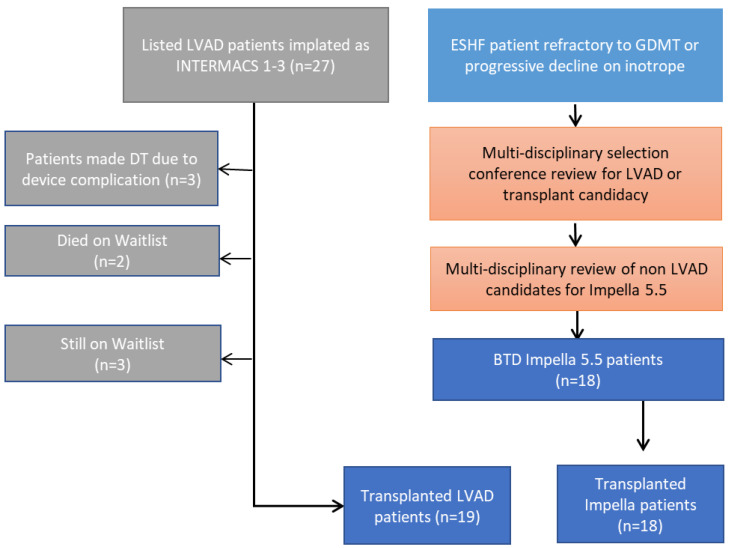
Study Flowchart.

Before non-emergent mechanical support placement, all patients were reviewed in our multi-disciplinary transplant selection committee for appropriateness—including review of anatomy, current heart failure functional class and stage, and risk for mortality while awaiting solid organ transplantation. Discussed patients had already been presented or listed for heart or heart–kidney transplantation or were accepted as hospital-to-hospital transfer in cardiogenic shock, necessitating escalation to temporary mechanical circulatory support (Figure 1)

### 2.2. Institutional Standard of Care

Our institutional practice includes patients’ medical, physical, and nutritional optimization as best tolerated in outpatient, with or without continuous home inotrope therapy. Subsequently, those patients with clinical or laboratory evidence of decline (e.g., increasing serum creatinine, progressive deterioration in functional status, inability to maintain a healthy weight, or intolerant of cardiopulmonary rehabilitation) and hemodynamic instability on right heart catheterization results are considered for admission and optimization. The standard of care for optimization at our institution for advanced heart failure patients includes the placement of a peripherally inserted central catheter (PICC) to monitor central venous pressures and record mixed venous saturation, thereby allowing us to calculate Fick cardiac output and index. We then devise a care plan based on the patient’s Stevenson profile. Finally, appropriate care, as determined by the treating physician, is undertaken. For those individuals that remain refractory to therapy, considerations for advanced treatments follow suit.

### 2.3. Patient Selection

Careful patient selection criteria must be used to apply appropriate support to patients. Patients implanted with an Impella 5.5 were patients with New York Heart Association (NYHA) class 3 or greater, American Heart Association (AHA) Stage D in need of or were already on inotrope therapy, with progression to dual inotropes or refractory to optimized guideline-directed medical therapy, and those with clinical testing (e.g., Cardiopulmonary exercise testing (CPET), Right heart catheterization (RHC), 6 min walk, etc.), consistent with the need for transplant evaluation fit the spectrum of patients that have been selected for the utilization of surgically placed Impella 5.5 support as a BTT. These patients were also deemed ineligible for LVAD by our heart team. The potential for optimizing patients and their end organ function with temporary mechanical circulatory support (tMCS) before declaring them unsuitable for orthotopic heart transplant (OHT) should be considered. Those deemed ineligible for OHT under the UNOS eligibility criteria and were LVAD candidates were implanted with a durable LVAD as destination therapy.

### 2.4. Multi-Disciplinary Impella Management

Monitoring after Impella support at our institution is standardized and consists of weekly trans-thoracic echocardiogram and daily chest radiograph with daily securement device assessment for patient comfort, as well as serum lactate dehydrogenase, renal profile, and complete blood counts. Additionally, patients have baseline pulmonary artery (PA) catheterization during their assessment for requiring advanced mechanical support if not already present before inotrope initiation. Most patients are monitored with an indwelling PA catheter for up to 96 h after Impella placement. Lastly, daily multi-disciplinary rounds comprising cardiovascular intensive care, transplant cardiology, cardiothoracic surgery, and physical therapy are implemented, and critical care nursing teams review patient maintenance of support and potential needs for escalating care.

## 3. Results

Our institution performed 87 heart and 4 heart–kidney transplants between October 2018 and February 2021. Forty-five (49%) patients utilized mechanical circulatory support as BTT, and twenty-seven of these (60%) patients used LVAD support (Table 1). Within the LVAD group, two patients (7%) died on the waitlist due to ischemic events, and three patients (11%) were converted to destination therapy due to device complication that rendered them ineligible for transplant, with three (11%) still on the waitlist. All eighteen Impella patients survived to transplant (100%) compared to nineteen bridged with durable LVAD (70%), *p* = 0.001. Of the 18 patients bridged with Impella 5.5, 17 were transplanted from donation after brain death (DBD) donors, and one received an organ from a donation after circulatory death (DCD) donor.

**Figure 2:**
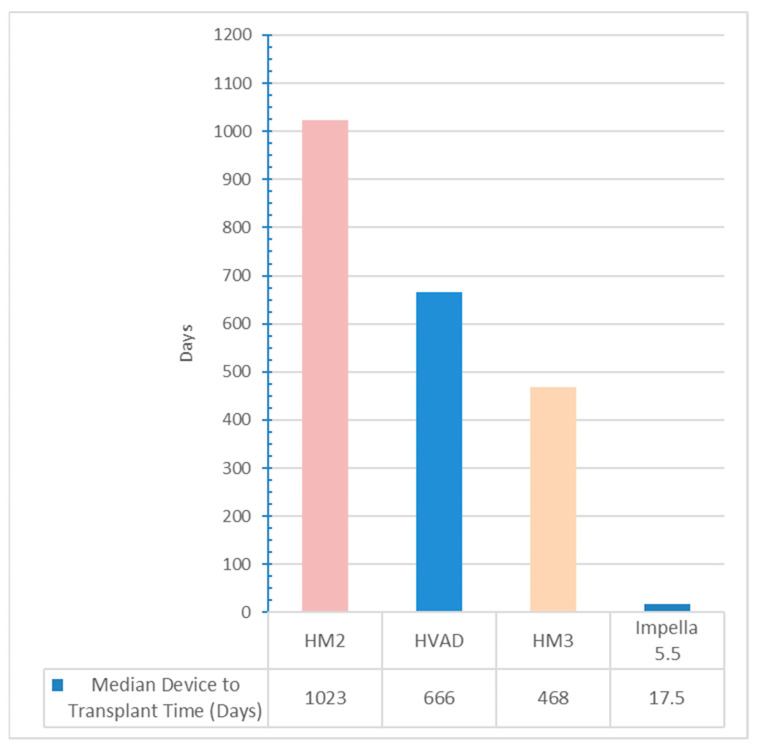
Comparison of the median device to transplant time (in days) between INTERMACS 1−3 patients from the time of device placement (*p* < 0.001 across all groups compared to Impella 5.5).

Patients in the Impella group were older, with a median age at transplant of 61 years (IQR 55–66) compared to 50 years (IQR 44–60) in the LVAD group, *p* = 0.019. The median duration from listing to transplant was significantly lower with Impella 5.5 compared to LVAD (35 days vs. 696 days, *p* < 0.001). Similarly, the median time from device placement to transplant was significantly lower with Impella 5.5 compared to LVAD (17.5 days vs. 666 days, *p* < 0.001); Figure 2.

Pre-transplant factors significantly associated with survival to transplant in a multivariate analysis include Impella use for BTT (*p* < 0.001) and INTERMACS profile at device implant (*p* < 0.001). Diabetic status (*p* = 0.594), etiology of cardiomyopathy (*p* = 0.533), and BMI at transplant (*p* = 0.291) were not significant factors in this model. HLA panel reactive antigens were not significantly different between the two groups. More Impella patients were UNOS Status 2 at transplant compared to the LVAD group (88% vs. 58%, *p* < 0.001) based on the current UNOS listing guidelines.

LVAD device-specific complications resulted in higher UNOS listing status at transplantation than Impella 5.5 (63% vs. 10%, *p* < 0.001). 12 of 19 LVAD patients (63%) were transplanted due to complications: 3 (25%) with RV failure on inotropes, 1 (8%) from stroke, 6 (50%) with driveline infection, and 2 (16%) with pump thrombosis (Table 1).

Donor factors were evaluated to neutralize potential bias in donor selection within our population of listed patients (Table 2). Donor sequence at acceptance was the only significantly different factor between the Impella 5.5 (4, IQR 1–6) compared to LVAD (5, IQR 1–32), *p* = 0.039.

BTT with Impella 5.5 had significantly shorter cardiopulmonary bypass times compared to the LVAD group—181 min vs. 219 min (*p* = 0.002)—and a lower post-bypass median vasoactive inotropic score of 7.9 in the Impella 5.5 group and 13 in the LVAD group (*p* = 0.003) (Table 3).

Post-transplant Impella vs. LVAD ICU length of stay (LOS) (4 vs. 6) and transplant-to-discharge LOS (12.5 vs. 14) were not significantly different (*p* = 0.495 and *p* = 0.498), respectively. 30-day readmissions were not significantly increased in the LVAD group compared to Impella 5.5 (3 vs. 1, *p* = 0.162).

There was no difference in 30-day survival between the two groups. Among LVAD-bridged patients, 1-year post-transplant survival was 100%, and 2-year survival was 79% (4 deaths). In the Impella 5.5-bridged group, 1-year survival was 94% and remained unchanged at 2 years.

## 4. Discussion

Increased utilization of temporary support devices as bridge therapy after the allocation changes in 2018 has led to concerns within the transplant community regarding the role of LVAD bridge therapy. As we continue to assess the impact of the difference in the UNOS allocation scheme, there has been developing evidence that current methods utilized to achieve the primary goal of heart transplantation with bridge therapy are essential [6,7]. In our literature assessment, dual-inotrope therapy strategies with continuous PA catheter monitoring or LVAD may no longer be sustainable or beneficial for our patient population. Utilizing Impella support as a bridge to recovery also provides time for patients with concomitant organ dysfunction and decompensated HFCS to be assessed for potential for renal and pulmonary hypertension recovery. Our previously presented data has demonstrated renal recovery in six patients awaiting heart and kidney transplant who eventually underwent heart-alone transplantation and did not require renal replacement therapy after transplantation. The focus on bridge therapy should be singular—survival to transplant with minimal complications in the pre-transplant and perioperative periods.

A study of 5746 patients comparing various durable LVADs with various Impella devices showed there was no statistical significance in the two cohorts where one-year mortality is concerned [8]. Our previously published data and other studies also corroborated this trend while highlighting the role in Impella 5.0 and 5.5 in enhanced LV unloading, reduced complications, early extubation, and ambulation, which may contribute to improved post-transplant outcomes [9,10,11]. Consistent with these findings, our cohort demonstrated that although patients receiving Impella 5.5 made up a sicker population than those receiving LVAD, given that they were older, more anemic, and with worse renal function, they had greater survival to transplant in the setting of shorter waitlist times and hemodynamic optimization without device-related complications. This translated to shorter intraoperative cardiopulmonary bypass time, less postoperative vasoplegia, and reduced intraoperative transfusions. Based on the data presented within this manuscript, the role of LVAD utilization as a bridge to transplant therapy is questioned by many of our patients, especially considering most patients receive organs after LVAD placement due to a complication. With a 26% death or de-listing rate with LVAD versus a 0% death or de-listing rate with Impella support, our data suggests we should reconsider the optimal bridge strategy in patients awaiting heart transplantation.

It is, however, important to recognize that durable LVADs play a crucial role in advanced heart failure care, particularly in those deemed ineligible to receive a heart transplant. In such patients, LVADs continue to provide improved quality of life, survival benefits, and extended bridging, and they continue to remain the standard of care for this subgroup. Our findings should not be interpreted as diminishing the role of LVADs but rather as highlighting an evolution in managing heart failure, one in which Impella 5.5 support offers superior short-term shock stabilization and outcomes under the current UNOS allocation guidelines [12].

## 5. Limitations

As a single-center retrospective study with a relatively small sample size, this is a hypothesis-generating data that is likely to be repeated at other large centers. Given the difference in patient profiles during device placement, we utilized INTERMACS as well as a vasoactive inotrope score to normalize the severity of illness for each patient at transplantation. UNOS Status in itself also helps with limiting the degree of difference in patient population based on bridge strategy. Hemodynamic data were captured as per the attending physician direction of PA catheter duration, and thus, due to the risk of infection or deep venous thrombosis risk in some patients, data was not available for the full 72 h after device placement in some instances.

## 6. Conclusions

As we demonstrated in our population, the optimal bridge to transplant strategy in the current era for INTERMACS Profile 1 to 3 patients with the UNOS allocation system may be using axillary mechanical support devices. This data may challenge current paradigms for the management of the sickest patients. Short- and long-term outcomes regarding renal failure after transplantation, donor-specific antibodies, and 1-year survival will be assessed in our cohort and presented in future publications. By mitigating risk and improving functional status before transplant, with fewer complications compared to the LVAD population, the role of axillary support should be further evaluated in a multi-center prospective fashion.

## Figures and Tables

**Table 1:** Baseline Characteristics.

	Impella 5.5 (n = 18)	LVAD (n = 27)	*p*-Value
**Age in years (Median, IQR)**	61 (54–66)	52 (44–62)	**0.023**
**Gender**			
• Male (n, %)	16 (89%)	19 (70%)	
**Blood Group**			
• A	4	10	
• B	1	2	
• AB	1	0	
• O	12	15	
**Baseline Labs and comorbidities**			
• Hematocrit (g/dL, Median, IQR)	29 (26–32)	34 (31–36)	**0.002**
• Body Mass Index (BMI) (Median, IQR)	28 (26–30)	30 (25–35)	0.169
• Creatinine (mg/dL, Median, IQR)	1.4 (1.3–1.8)	1.4 (1.1–1.6)	**0.04**
• Diabetic status (n, %)	7 (39%)	8 (30%)	0.268
**Etiology**			
• Ischemic	6 (33%)	11 (41%)	
• Non-ischemic	11 (61%)	14 (52%)	
• Congenital	1 (5%)	1 (3%)	
• LVAD pump exchange	0	1 (3%)	
**Average (Min–max) HLA Class 1 PRA %**	3 (0–21)	15 (0–100)	**0.014**
**Average (Min–max) HLA Class 2 PRA %**	17 (0–100)	7 (0–47)	0.119
**Mechanical support device (%)**			
• HeartWare HVAD	0	13 (48%)	
• HeartMate 3	0	10 (37%)	
• HeartMate 2	0	4 (15%)	
• Impella 5.5	18 (100%)	0	
**Outcome (%)**			
• Transplanted	18 (100%)	19 (70%)	**0.001**
**INTERMACS Score at Implant**			
• 1	-	8	**0.027**
• 2	18	9	
• 3	-	2	
**UNOS Status at Transplant**			
• 1	2	1	**<0.001**
• 2	13	3	
• 2e	3	9	
• 3	0	3	
• 4	0	3	
**Complication resulting in status upgrade**	1 (5%)	12 (63%)	
• RV Failure	1 (100%)	3 (25%)	**<0.001**
• Stroke	0	1 (8%)	
• Driveline infection	0	6 (50%)	
• Pump thrombosis	0	2 (16%)	
• Remain on waitlist	0	3 (11%)	
• Died on waitlist	0	2 (7%)	

**Table 2:** Baseline Donor Characteristics.

	Impella 5.5 (n = 18)	LVAD (n = 19)	*p* Value
**Donor age (Median, IQR)**	32 (28–36)	29 (22–32)	0.078
**Donor Gender**			
• Male	17 (94%)	16 (84%)	
• Gender Mismatch	3	2	
**Donor Distance (Median, IQR)**	336 (200–586)	273 (113–499)	0.087
**Offer Sequence (Median, IQR)**	4 (1–6)	5 (1–32)	**0.039**
**Downtime in minutes (Median, IQR)**	0 (0–46)	0 (0–51)	0.355
**Initially reported LVEF % (Median, IQR)**	59 (55–65)	60 (56–65)	0.341
**PHS increased risk**	3	5	
**Hepatitis C-positive donor**	0	2	
**DBD Donor**	17	19	
**DCD Donor**	1	0	

**Table 3:** Peri-, Intra-, and Post-operative Data.

Parameter (Median, IQR)	Impella 5.5 (n = 18)	LVAD (n = 19)	*p* Value
Listing to Transplant (days)	35 (15–75.25)	696 (298–750)	**<0.001**
Device to Transplant (days)	18 (11–27)	666 (544–914)	**<0.001**
Cardiopulmonary bypass time (minutes)	181 (156–197)	219 (191–244)	**0.002**
Cold ischemic time (minutes)	222 (201–239)	230 (198–251)	0.30
Packed red blood cell (of units)	4 (3–5)	4 (3.5–6.5)	0.204
Fresh frozen plasma (mL)	625 (500–1000)	800 (500–1125)	0.086
Cryoprecipitate (mL)	110 (0–200)	200 (200–300)	**0.011**
Autologous transfusion (mL)	675 (450–900)	1125 (1013–1050)	**0.001**
Platelets (mL)	350 (250–500)	675 (425–1000)	**0.005**
Immediate post-operative vasoactive inotrope score	7.9 (5–11.9)	13 (9–16.8)	**0.003**
ICU length of stay (days)	4 (3.25–6.75)	6 (4.5–8.5)	0.495
Post-Transplant-to-discharge duration (days)	12.5 (11.3–14.8)	14 (12–21)	0.498

## Data Availability

The data presented in this study are available on request from the corresponding author.

## Abbreviations

The following abbreviations are used in this manuscript:

BTT	bridge to transplant
DBD	donation after brain death
DCD	donation after circulatory death
HFCS	heart failure cardiogenic shock
LOS	length of stay
LVAD	left ventricular assist device
MCS	mechanical circulatory support
tMCS	temporary mechanical circulatory support
UNOS	united network for organ sharing
